# Radiographs in screening for sacroiliitis in children: what is the value?

**DOI:** 10.1186/s13075-018-1642-8

**Published:** 2018-07-11

**Authors:** Pamela F. Weiss, Rui Xiao, Timothy G. Brandon, David M. Biko, Walter P. Maksymowych, Robert G. Lambert, Jacob L. Jaremko, Nancy A. Chauvin

**Affiliations:** 1 Department of Pediatrics, Division of Rheumatology, Children’s Hospital of Philadelphia, Philadelphia, PA USA; 2 Department of Radiology, Children’s Hospital of Philadelphia, Philadelphia, PA USA; 3 Center for Pediatric Clinical Effectiveness (CPCE), Children’s Hospital of Philadelphia, Philadelphia, PA USA; 4 Center for Clinical Epidemiology and Biostatistics, Perelman School of Medicine at the University of Pennsylvania, Philadelphia, PA USA; 5 Department of Medicine, University of Alberta, Edmonton, AB Canada; 6 Department of Radiology and Diagnostic Imaging, University of Alberta, Edmonton, AB Canada; 7 Canadian Research and Education (CaRE) Arthritis Organization, Edmonton, AB Canada; 8 The Children’s Hospital of Philadelphia, Roberts Center for Pediatric Research, 2716 South Street, Room 11121, Philadelphia, PA 19146 USA

**Keywords:** Radiograph, Magnetic resonance imaging, Sacroiliitis, Juvenile spondyloarthritis, Misclassification

## Abstract

**Background:**

We aimed to evaluate the diagnostic utility of pelvic radiographs versus magnetic resonance imaging (MRI) of the sacroiliac joints in children with suspected sacroiliitis.

**Methods:**

This was a retrospective cross-sectional study of children with suspected or confirmed spondyloarthritis who underwent pelvic radiograph and MRI within 6 months of one another. Images were scored independently by five raters. Interrater reliability was calculated using Fleiss’s kappa coefficient (κ). Test properties of radiographs for depiction of sacroiliitis were calculated using MRI global sacroiliitis impression as the reference standard.

**Results:**

The interrater agreement for global impression was κ = 0.34 (95% CI 0.19–0.52) for radiographs and κ = 0.72 (95% CI 0.52–0.86) for MRI. Across raters, the sensitivity of radiographs ranged from 25 to 77.8% and specificity ranged from 60.8 to 92.2%. Positive and negative predictive values ranged from 25.9 to 52% and from 82.7 to 93.9%, respectively. The misclassification rate ranged from 6 to 17% for negative radiographs/positive MRI scans and from 48 to 74% for positive radiographs/negative MRI scans. When the reference standard was changed to structural lesions consistent with sacroiliitis on MRI, the misclassification rate was higher for negative radiographs/positive MRI scans (9–23%) and marginally improved for positive radiographs/negative MRI scans (33–52%).

**Conclusion:**

Interrater reliability of MRI was superior to radiographs for global sacroiliitis impression. Misclassification for both negative and positive radiographs was high across raters. Radiographs have limited utility in screening for sacroiliitis in children and result in a significant proportion of both false negative and positive findings versus MRI findings.

## Background

Juvenile spondyloarthritis (SpA) is a term that encompasses a group of conditions characterized by inflammatory arthritis, enthesitis, HLA-B27 positivity, acute anterior uveitis, inflammatory bowel disease, and psoriasis. The arthritis of juvenile SpA (JSpA) can be peripheral or axial (sacroiliac joints or spine). While the diagnosis of peripheral arthritis can typically be made by clinical examination, confirmation of sacroiliitis often requires imaging. Prior studies have shown that tenderness to palpation and physical examination maneuvers such as the flexion abduction external rotation (FABER) hip test have low sensitivity and specificity for sacroiliitis using magnetic resonance imaging (MRI) as the reference standard [1]. For historical reasons, radiographs are currently the gold standard for making the diagnosis of ankylosing spondylitis and are frequently a prerequisite for obtaining an MRI study under many insurance plans in the United States. Radiographs, however, only show bony damage and are not sensitive enough to detect early disease or incremental changes over short periods of time [2]. Given the relatively short disease duration and rare occurrence of ankylosis in children [1, 3, 4], the value of radiographs at the time of diagnosis or in evaluation of suspected early inflammatory sacroiliitis in children is unclear. The practice of routinely obtaining radiographs may cause unnecessary radiation exposure and result in early cases of sacroiliitis going undetected and untreated if MRI is not subsequently performed [1]. Misdiagnosis may also result in inappropriate therapy.

MRI has become increasingly utilized to detect inflammation in the sacroiliac joints (SIJs) before changes are apparent on radiographs. The Assessment of SpondyloArthritis International Society (ASAS) classification criteria for axial spondyloarthritis specifically include MRI evidence of inflammation in the sacroiliac joints in the criteria for adult SpA [5]. Several studies have shown the value of MRI in evaluation of JSpA [1, 6–8], but only one small study has directly evaluated the diagnostic utility of radiography versus MRI for children with SpA [7]. Concordant with other studies which show unequivocal superiority of MRI over radiographs for detection of active disease [9–11], the positive likelihood ratio (LR+) for a clinical diagnosis of SpA was much higher for MRI findings than radiographic findings in that small study, especially for erosions (LR+ = 6.7 vs 3.5) and global impression (LR+ = 9.4 vs 4.4) [7]. However, that study had a small sample size and a high frequency of abnormalities reported in controls; 55% and 20% had sclerosis and erosions by radiograph, respectively. This may have been due to use of oversensitive criteria for these radiographic findings [12].

The objective of this project was to more fully evaluate the accuracy of radiographs to detect sacroiliitis in children using global impression of the MRI study as the reference standard. Our working hypotheses were that radiographs do not add incremental value to the MRI examination of the sacroiliac joint and that the test properties of radiographs are sufficiently low that follow-up MRI is needed in most cases.

## Methods

### Human subject protection

The protocol for the conduct of this study was reviewed and approved by the Children’s Hospital of Philadelphia Committee for the Protection of Human Subjects (IRB 16-013013).

### Study population

This was a retrospective cross-sectional study of all children with suspected or confirmed JSpA who underwent both pelvic radiograph and MRI separated by no more than 6 months between January 2012 and May 2016. Eligible children were ages 6–18 years at the time of clinical care and had the following imaging protocols performed at our institution: anterioposterior (AP) view of the pelvis or dedicated radiographs and MRI of the sacroiliac joints that included coronal oblique T1 and STIR sequences performed at either 1.5 or 3 Tesla. All MRI assessments were made using noncontrast sequences. Demographic characteristics and indications for imaging were abstracted from the electronic medical record and the imaging studies were obtained from the picture archiving and communication system (PACS).

### Evaluation of imaging studies

All scoring exercises were completed within a web-based environment (CaREArthritis.com) or Research Electronic Data Capture (REDCap) [13]. REDCap is a secure, web-based application designed to support data capture for research studies. Four raters were musculoskeletal radiologists (DMB, RGL, JLJ, NAC) and one rater was an adult rheumatologist with SIJ imaging expertise (WPM). All images were reviewed in random order and blinded to clinical details. All raters have had extensive training in the interpretation of pelvic radiographs and MRI.

Each radiograph was assessed for erosions, sclerosis, joint space narrowing, joint space widening, and ankylosis. Each rater indicated whether the radiograph was globally representative of sacroiliitis (yes or no) and rated confidence in global impression (ordinal scale − 4 to 4 with anchors of “definitely no” and “definitely yes”). Erosion was defined as a cortical irregularity along the articular surface of the bone. Sclerosis was defined as increased subchondral bone density compared to the subchondral bone density in the hips/spine. Ankylosis was defined as complete obliteration of the joint space with contiguous bone between the sacrum and ilium. Joint space narrowing and widening were determined subjectively as decreased or increased width of the joint space. The presence of each lesion was recorded as occurring in the left or right joint, with an additional specification of quadrant location being made for erosion and sclerosis.

Each MRI study was evaluated for active inflammatory lesions (bone marrow edema, capsulitis, SIJ effusion, enthesitis outside of the SIJ) and structural lesions (erosion, sclerosis, fat metaplasia, backfill, ankylosis). Inflammation was assessed using the CareArthritis platform and the Spondyloarthritis Research Consortium of Canada (SPARCC) SIJ Inflammation Score (SIS) scoring module. Reliability of the SPARCC SIS has been demonstrated in the pediatric population [14, 15]. Details about the platform and scoring have been published previously [16]. All raters previously completed calibration exercises for the SPARCC SIS and SSS with acceptable reliability (intraclass correlation (ICC) ≥ 0.8) [14, 15]. The presence or absence of marrow edema was scored for each joint quadrant (total score per slice 0–8). Marrow edema was deemed present if the intensity was the same or greater than the presacral veins and depth ≥ 1 cm, and was scored dichotomously for each sacroiliac joint. Positive bone marrow edema findings were defined in accordance with the ASAS criteria (bone marrow edema in two or more locations on a single MRI slice or bone marrow edema on two consecutive MRI slices). The ASAS MRImagine consensus-based eCRF for recording MRI data was used to capture the following: rater global impression of acute/active inflammatory lesions compatible with sacroiliitis (yes/no), rater global impression of structural lesions typical of axial SpA, confidence in that assessment (ordinal scale − 4 to 4 with anchors of “definitely no” and “definitely yes”), capsulitis (yes/no), SIJ effusion (yes/no), and enthesitis outside of the SIJ (yes/no) [17, 18].

Structural lesions on MRI (erosion, sclerosis, fat metaplasia, backfill, ankyloses) were assessed using the CareArthritis platform and the SPARCC SIJ structural score (SSS) scoring module. Reliability of the SPARCC SSS has been demonstrated in the pediatric population [19].

### Statistical analysis

Subject demographic characteristics and raters’ assessments of lesions were summarized by frequencies and percentages or medians and interquartile ranges (IQR). In order to compare radiograph and MRI assessment for lesions, all MRI scoring was dichotomized. Interrater agreement was assessed using Fleiss’s kappa statistic with bootstrap confidence intervals [20], with agreement interpreted as poor ≤ 0.40, fair 0.41–0.59, good 0.60–0.74, and excellent ≥ 0.75 [21]. Sensitivity, specificity, positive predictive value, and negative predictive value were calculated to assess the performance of radiographs in identifying sacroiliitis (global impression “yes”) using MRI global impression of active sacroiliitis (“yes”) as the reference standard. We also conducted two analogous analyses in which the reference standard was altered: to assess performance of radiographs in identifying sacroiliitis using MRI global impression of structural lesions consistent with sacroiliitis (global impression “yes”) as the reference standard; and to assess performance of radiographs in identifying sacroiliitis using MRI global impression of active sacroiliitis (global impression “yes”) *or* structural lesions consistent with sacroiliitis (global impression “yes”) as the reference standard. All analyses were performed using Stata 14.2 (2015, Stata Statistical Software Release 14; StataCorp. LP, College Station, TX, USA).

## Results

### Subjects

**Fig. 1:**
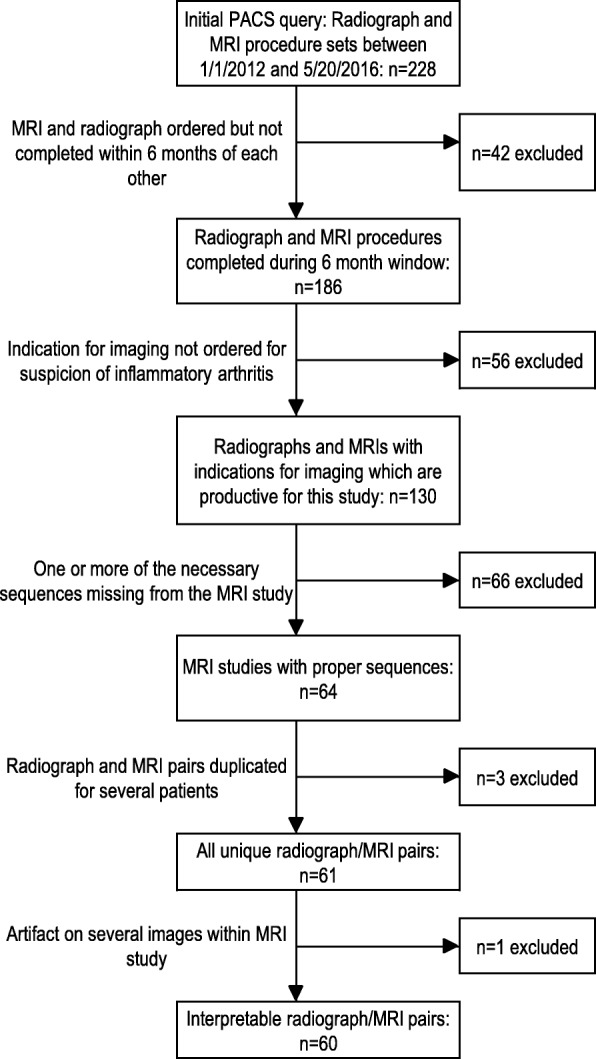
Sample size flow chart for identification of subjects meeting inclusion criteria. PACS picture archiving and communication system, MRI magnetic resonance imaging

A total of 228 children had both a radiograph and an MRI ordered during the study window; 60 pairs of images met our inclusion criteria (Fig. 1). The median time between studies was 0 days (IQR 0–10 days). In 41 (68.3%) cases, radiograph and MRI occurred on the same day, 16 (26.7%) cases had a radiograph that preceded the MRI, and the remaining three (5.0%) cases had an MRI prior to the radiograph. The primary indication for imaging in our study population was inclusion in a prior study evaluating the prevalence of sacroiliitis in patients newly diagnosed with JSpA [1] (*n* = 38, 63.3%). The remaining subjects were imaged for complaints of back (23.3%) or hip (3.3%) pain, limited range of motion (1.7%), or as follow-up for a previous sacroiliitis diagnosis (8.3%). Demographics are presented in Table 1. Half of the subjects were male and the median age at time of radiograph was 14.4 years (IQR 11.9–16.6 years). Eighty percent of subjects were Caucasian and 12% were African American.

**Table 1:** Subject characteristics

	Frequency (%)/median (IQR) (*n* = 60)
Age at radiograph (years)	14.4 (11.9–16.6)
Age at MRI (years)	14.6 (11.9–16.7)
Time between studies (months)	0 (0–0.3)
Sex, male	30 (50%)
Race	
White/Caucasian	48 (80%)
Black/African American	7 (12%)
Asian	1 (2%)
Other	4 (7%)

*IQR* interquartile range, *MRI* magnetic resonance imaging

### Radiographs

Fifty-four (90%) subjects had a single AP pelvic radiograph, three subjects had AP and frogleg sacroiliac radiographs, and three subjects had AP and bilateral oblique dedicated sacroiliac radiographs performed*.* Fourteen (23.3%) radiographs were read as abnormal by three or more raters. Of those radiographs with at least three raters reporting abnormal findings, all raters called the study abnormal in five (35.7%) cases. The most common radiographic findings across 300 reads completed by the five raters were sclerosis (29%), erosion (22%), and joint space widening (14%). The interrater agreement for global impression and all lesions assessed by radiograph are presented in Table 2. The interrater agreement for global impression of active sacroiliitis was poor (κ = 0.34, 95% CI 0.19–0.52). The interrater agreement for each radiographic lesion was either poor or fair. The interrater reliability could not be determined for ankylosis due to low prevalence. One hundred and seventy three of 300 (57.7%) assessments of active arthritis had a confidence level of “definitely yes” or “definitely no”.

**Table 2:** Radiologist findings and agreement for radiograph and MRI

	Frequency (%)					
	Rater 1	Rater 2	Rater 3	Rater 4	Rater 5	Kappa (95% CI)
Radiograph						
Global impression of sacroiliitis	8 (1)	8 (13)	27 (45)	17 (28)	25 (42)	0.34 (0.19–0.52)
Erosion	7 (12)	6 (10)	25 (42)	12 (20)	15 (26)	0.42 (0.22–0.6)
Sclerosis	8 (13)	8 (13)	21 (35)	19 (32)	32 (53)	0.35 (0.19–0.52)
Ankylosis	0 (0)	0 (0)	2 (3)	3 (5)	4 (7)	–
JSN	1 (2)	1 (2)	10 (17)	18 (30)	8 (13)	0.11 (0.03–0.2)
JSW	7 (12)	5 (8)	13 (22)	10 (17)	8 (13)	0.4 (0.23–0.57)
MRI						
Global impression of acute/active sacroiliitis^a^	12 (20)	9 (15)	9 (15)	10 (17)	17 (28)	0.72 (0.52–0.86)
BME	10 (17)	9 (15)	12 (20)	10 (17)	16 (27)	0.78 (0.58–0.9)
Capsulitis	6 (10)	2 (3)	0 (0)	1 (2)	7 (12)	0.21 (−0.02–0.46)
Enthesitis outside of SIJ	6 (10)	3 (5)	1 (2)	0 (0)	4 (7)	0.25 (− 0.01–0.52)
SIJ effusion	1 (2)	3 (5)	7 (12)	11 (18)	17 (28)	0.19 (0.05–0.34)
Global impression of structural chronic^b^ lesions consistent with sacroiliitis	16 (27)	9 (15)	12 (20)	13 (22)	19 (32)	0.58 (0.39–0.74)
Erosion	14 (23)	9 (15)	9 (15)	13 (22)	9 (15)	0.64 (0.48–0.81)
Sclerosis	4 (7)	4 (7)	6 (10)	12 (20)	36 (60)	0.15 (0.01–0.31)
Backfill	11 (18)	4 (7)	6 (10)	4 (7)	6 (10)	0.59 (0.24–0.81)
Fat metaplasia	3 (5)	1 (2)	2 (3)	1 (2)	5 (8)	0.52 (−0.02–0.93)
Ankylosis	0 (0)	1 (2)	0 (0)	1 (2)	1 (2)	–

*BME* bone marrow edema, *CI* confidence interval, *JSN* joint space narrowing, *JSW* joint space widening, *MRI* magnetic resonance imaging, *SIJ* sacroiliac joint

^a^Acute/active inflammatory lesions meeting the Assessment of Spondyloarthritis International Society definition of a positive MRI scan of the sacroiliac joints

^b^Structural chronic lesions refer to the clear presence of typical findings such as sclerosis, erosion, fatty lesions, bone bridges, and ankyloses

### Magnetic resonance imaging

Ten (16.7%) pelvic MRI scans were considered indicative of active sacroiliitis by at least three of the raters. Of these MRI studies with at least three raters reporting active sacroiliitis, all raters called the study abnormal in eight (80%) cases. Across all studies, periarticular bone marrow edema was noted in 19%. The most commonly detected structural MRI lesions across all studies were sclerosis (21%) and erosion (18%). The interrater agreement for global impression and all lesions assessed by MRI are presented in Table 2. The kappa value for global MRI impression of active sacroiliitis was good (κ = 0.72, 95% CI 0.52–0.86). The interrater agreement for bone marrow edema, the key lesion for classification according to the ASAS criteria, was excellent (κ = 0.78, 95% CI 0.58–0.9) [22]. The kappa values for other lesions consistent with active sacroiliitis (capsulitis, enthesitis, SIJ effusion) were low (κ ≤ 0.25). The interrater agreement for global impression of structural lesions consistent with sacroiliitis was fair (κ = 0.58, 95% CI 0.39–0.74). The interrater agreement for each of the structural lesions assessed on MRI were fair to good with the exception of sclerosis (κ = 0.15) and ankylosis, which again could not be assessed secondary to the low prevalence of lesions. Two hundred and thirty of 300 (76.6%) assessments had a confidence level of “definitely yes or definitely no”.

### Radiograph versus MRI

**Fig. 2:**
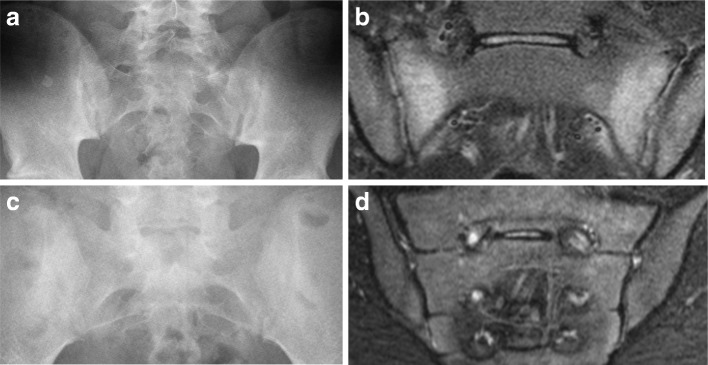
Samples of discordant radiograph and MRI overall impressions of presence/absence of sacroiliitis. **a** and **b**) 16-year-old HLA-B27+ male with 2 months of hamstring, gluteal, and low back pain. (**a**) Radiograph – normal (no sacroiliitis) by 5/5 raters; two raters noted sclerosis. (**b**) MRI STIR – abnormal (sacroiliitis present) rated by 5/5 raters; bilateral sacral subchondral bone marrow edema is clearly present. Two raters reported a positive erosion finding and 3 raters reported a positive sclerosis finding. (**c** and **d**) 13-year-old HLA-B27 negative female with lower and mid-back pain with accompanying morning stiffness, acute uveitis, and multiple tender entheses. (**c**) Radiograph – rated abnormal (sacroiliitis present) by 5/5 raters; two raters reported a positive finding for erosion, two reported a finding of joint space narrowing, and all five raters reported sclerosis. (**d**) MRI STIR – rated as normal (no sacroiliitis) by 5/5 raters; no abnormalities (erosion, sclerosis, fat metaplasia, ankylosis, or backfill) were reported on MRI. Radiologist raters were blinded to patient clinical details and are included here to provide the reader with relevant medical history

Table 3 presents the performance of radiographs in identifying sacroiliitis (global impression “yes”) using MRI global impression of active sacroiliitis (“yes”) as the reference standard. The sensitivity of radiographs for sacroiliitis on MRI ranged from 25 to 77.8% and specificity ranged from 60.8 to 92.2% across raters. Positive and negative predictive values ranged from 25.9 to 52% and from 82.7 to 93.9%, respectively. Across raters, the misclassification rate ranged from 6 to 17% for negative radiographs/positive MRI scans and from 48 to 74% for positive radiographs/negative MRI scans. Examples of agreement from 5/5 raters on positive radiograph/negative MRI and negative radiograph/positive MRI are shown in Fig. 2. Of the 51 cases across raters where positive radiographs were paired with negative MRI scans, 12 (23.5%) of the MRI scans showed structural lesions in the absence of findings consistent with active inflammatory sacroiliitis. The reported structural abnormalities were erosion (*n* = 6), sclerosis (*n* = 7), backfill (*n* = 1), and fat metaplasia (*n* = 1). Of the five cases where all raters’ impression on radiograph was congruent with sacroiliitis, two of those cases were found to be normal on MRI by all raters. Eight cases had total agreement on global impression of sacroiliitis “yes” among raters on MRI, five of which were paired with a sacroiliitis impression “yes” from three or more raters on the corresponding radiographs.

**Table 3:** Test properties of radiograph for detection of active inflammatory sacroiliitis on MRI

	Rater 1	Rater 2	Rater 3	Rater 4	Rater 5
Percentage (95% CI)					
Sensitivity	25.0 (5.5–57.2)	44.4 (13.7–78.8)	77.8 (40.0–97.2)	70.0 (34.8–93.3)	76.5 (50.1–93.2)
Specificity	89.6 (77.3–96.5)	92.2 (81.1–97.8)	60.8 (46.1–74.2)	80.0 (66.3–90.0)	72.1 (56.3–84.7)
PPV	37.5 (8.5–75.5)	50.0 (15.7–84.3)	25.9 (11.1–46.3)	41.2 (18.4–67.1)	52.0 (31.3–72.2)
NPV	82.7 (69.7–91.8)	90.4 (79.0–96.8)	93.9 (79.8–99.3)	93.0 (80.9–98.5)	88.6 (73.3–96.8)
Frequency (%)					
Radiograph–^a^					
Radiograph–/MRI–	43 (83)	47(90)	31 (94)	40 (93)	31 (89)
Radiograph–/MRI+	9 (17)	5 (10)	2 (6)	3 (7)	4 (11)
Radiograph+ ^a^					
Radiograph+/MRI–	5 (63)	4 (50)	20 (74)	10 (59)	12 (48)
Radiograph+/MRI+	3 (37)	4 (50)	7 (26)	7 (41)	13 (52)

Kappa value interpretations: ≤ 0.40 poor agreement, 0.41–0.59 fair agreement, 0.60–0.74 good agreement, and ≥ 0.75 excellent agreement [21]. For sensitivity, specificity, PPV, and NPV calculations, positive radiograph defined as global impression of sacroiliitis (yes) and reference standard was MRI global impression of active sacroiliitis (yes)

*CI* confidence interval, *MRI* magnetic resonance imaging, *NPV* negative predictive value, *PPV* positive predictive value

^a^MRI and radiograph + or – defined as global impression of sacroiliitis (yes/no)

Table 4 presents the performance of radiographs in identifying sacroiliitis (global impression “yes”) when the reference standard was changed to MRI global impression of structural lesions consistent with sacroiliitis (“yes”). Across raters, the sensitivity of radiographs for structural lesions consistent with sacroiliitis on MRI ranged from 25 to 75% and specificity ranged from 70.7 to 90.9%. Positive and negative predictive values ranged from 37.5 to 52% and from 76.9 to 90.9%, respectively. Across raters, the misclassification rate ranged from 9.1 to 23.1% for negative radiographs/positive MRI scans and from 48.0 to 66.7% for positive radiographs/negative MRI scans. When the reference standard was changed to MRI global impression of active *or* structural lesions consistent with sacroiliitis on MRI, the test properties and misclassification rate were similar (Table 5).

**Table 4:** Test properties of radiograph for detection of structural lesions consistent with sacroiliitis on MRI

	Rater 1	Rater 2	Rater 3	Rater 4	Rater 5
Percent (95% CI)					
Sensitivity	25.0 (7.3–52.4)	33.3 (7.5–70.1)	75.0 (42.8–94.5)	53.8 (25.1–80.8)	68.4 (43.4–87.4)
Specificity	90.9 (78.3–97.5)	90.2 (78.6–96.7)	62.5 (47.4–76.0)	78.7 (64.3–89.3)	70.7 (54.5–83.9)
PPV	50.0 (15.7–84.3)	37.5 (8.5–75.5)	33.3 (16.5–54.0)	41.2 (18.4–67.1)	52.0 (31.3–72.2)
NPV	76.9 (63.2–87.5)	88.5 (76.6–95.6)	90.9 (75.7–98.1)	86.0 (72.1–94.7)	82.9 (66.4–93.4)
Frequency (%)					
Radiograph–^a^					
Radiograph–/MRI–	40 (76.9)	46 (88.5)	30 (90.9)	37 (86)	29 (82.9)
Radiograph–/MRI+	12 (23.1)	6 (11.5)	3 (9.1)	6 (14)	6 (17.1)
Radiograph+^a^					
Radiograph+/MRI–	4 (50)	5 (62.5)	18 (66.7)	10 (58.8)	12 (48)
Radiograph+/MRI+	4 (50)	3 (37.5)	9 (33.3)	7 (41.2)	13 (52)

Kappa value interpretations: ≤ 0.40 poor agreement, 0.41–0.59 fair agreement, 0.60–0.74 good agreement, and ≥ 0.75 excellent agreement [21]. For sensitivity, specificity, PPV, and NPV calculations, positive radiograph defined as global impression of sacroiliitis (yes) and reference standard was MRI global impression of structural lesions consistent with sacroiliitis (yes)

*CI* confidence interval, *MRI* magnetic resonance imaging, *NPV* negative predictive value, *PPV* positive predictive value

^a^MRI and radiograph + or – defined as global impression of sacroiliitis (yes/no)

**Table 5:** Test properties of radiograph for detection of active *or* structural lesions typical of sacroiliitis on MRI

	Rater 1	Rater 2	Rater 3	Rater 4	Rater 5
Percent (95% CI)					
Sensitivity	22.2 (6.4–47.6)	33.3 (9.9–65.1)	75.0 (42.8–94.5)	57.1 (28.9–82.3)	64.0 (42.5–82.0)
Specificity	90.5 (77.4–97.3)	91.7 (80.0–97.7)	62.5 (47.4–76.0)	80.4 (66.1–90.6)	74.3 (56.7–87.5)
PPV	50.0 (15.7–84.3)	50.0 (15.7–84.3)	33.3 (16.5–54.0)	47.1 (23.0–72.2)	64.0 (42.5–82.0)
NPV	73.1 (59.0–84.4)	84.6 (71.9–93.1)	90.9 (75.7–98.1)	86.0 (72.1–94.7)	74.3 (56.7–87.5)
Frequency (%)					
Radiograph–^a^					
Radiograph–/MRI–	38 (73.1)	44 (84.6)	30 (90.9)	37 (86)	26 (74.3)
Radiograph–/MRI+	14 (26.9)	8 (15.4)	3 (9.1)	6 (14)	9 (25.7)
Radiograph +					
Radiograph+/MRI–	4 (50)	4 (50)	18 (66.7)	9 (52.9)	9 (36)
Radiograph+/MRI+	4 (50)	4 (50)	9 (33.3)	8 (47.1)	16 (64)

Kappa value interpretations: ≤ 0.40 poor agreement, 0.41–0.59 fair agreement, 0.60–0.74 good agreement, and ≥ 0.75 excellent agreement [21]. For sensitivity, specificity, PPV, and NPV calculations, positive radiograph defined as global impression of sacroiliitis (yes) and reference standard was MRI global impression of active inflammatory (yes) *or* structural lesions consistent with sacroiliitis (yes)

*CI* confidence interval, *MRI* magnetic resonance imaging, *NPV* negative predictive value, *PPV* positive predictive value

^a^MRI and radiograph + or – defined as global impression of sacroiliitis (yes/no)

In order to examine whether the test properties of radiographs for the detection of inflammatory sacroiliitis were robust to whether the child was acutely symptomatic or not, we performed a sensitivity analysis by excluding those children who were imaged as part of a prior study and were asymptomatic. In the restricted sample of 42 children, the sensitivity of radiographs ranged from 20 to 77.8% and the specificity ranged 69.7 to 90.6%.

## Discussion

This is a systematic analysis to compare the utility of radiographs to MRI in the evaluation of suspected sacroiliitis. Interrater reliability and confidence of MRI were superior to radiographs for global impression of sacroiliitis. The rates of misclassification were high, with false positive radiographs occurring more frequently than false negative radiographs. Radiographs do remain indispensable for identification of lesions that may be on the differential of pediatric lower back pain including osteomyelitis, septic arthritis, chronic recurrent multifocal osteomyelitis, tumor, and fracture. Our results, however, indicate that radiographs have limited utility in screening for inflammatory or structural lesions consistent with sacroiliitis in children and result in a significant proportion of both false negative and positive findings.

Several limitations should be considered while interpreting our findings. First, our sample size was limited to 60 sets of radiographs and MRI scans. The number of children who had imaging performed at our institution during the study timeframe was much larger, but many did not have both radiograph and MRI performed within 6 months of each other. Further, MRI at our institution prior to 2012 did not routinely include coronal oblique sacral sequences, which we consider vital for adequate assessment of the sacroiliac joints. Nevertheless, even with our limited sample size we were able to identify significant shortcomings in the use of radiographs in children. Second, this was a retrospective study using sets of images that could have been obtained at any point in the child’s disease course. Perhaps the utility of radiographs is higher with longer disease duration or older age and more closely matches the utility seen in adults in these cases of prolonged disease exposure. The vast majority of children, however, have relatively short duration of symptoms and disease at the time imaging is ordered. We think our data reflect the typical use of radiographs for routine practice in the evaluation of the sacroiliac joints in children with both suspected and established JSpA. Third, some of the subjects had imaging performed shortly after spondyloarthritis diagnosis as part of a prior study. Our sensitivity analysis investigating this limitation using a sample restricted to those patients imaged specifically because of pain (no prior research subjects) demonstrated that our range of estimates of the radiograph test properties did not vary between the full and restricted samples. Fourth, since this was not a prospective study, there was no imaging protocol and there were differences in imaging sequences obtained. All children had at least a single AP view radiograph performed, by study design, but some children had dedicated films with multiple views. It is possible that the sensitivity and specificity of a single view radiograph is inferior to multiple views. However, in a study of adults with seronegative spondyloarthritis, there was excellent agreement (greater than 86% for both left and right joints) between AP views and AP plus oblique projections [23]. All MRI sequences, by study design, included coronal oblique views on both T1 and T2 sequences to ensure adequate visualization of the sacroiliac joints. There are no strict measurements for depiction of sacroiliac joint effusions on MRI in children. The presence or absence of abnormality is, therefore, a subjective call which is likely the reason why these lesions had low agreement between the raters. Until there are sufficient normative data in children, definitive characterization of abnormal amounts of fluid for the sacroiliac joints will remain difficult. Fifth, our study results are not applicable to parts of the world in which a MRI scan is difficult to obtain. In these areas, radiographs remain the only option for screening. Sixth, unlike the other prior study [7], this imaging-only study uses MRI findings as the gold standard for sacroiliitis, without direct use of an external reference standard. Pathologic confirmation of sacroiliitis, for example by biopsy, is rarely feasible, and clinical diagnosis of spondyloarthropathy is complex; determining the relation between MRI findings of sacroiliitis and a clinical diagnosis of spondyloarthritis or juvenile idiopathic arthritis is beyond the scope of this work.

A few findings from this study warrant additional consideration. First, the specificity of radiographs for detection of sacroiliitis using MRI as the reference standard was relatively high (60.8–92.2) with slightly higher negative predictive values (82.7–93.9). However, when we looked at the children who actually had sacroiliitis, the majority had normal radiographs. The positive and negative predictive values and rates of misclassification were very similar when the reference standard was changed to structural lesions on MRI. This means that if radiographs remain the gold standard for screening, then almost all cases of sacroiliitis, even if symptoms prompted the imaging, would be missed. If we apply the concept of an early treatment window, as has been shown in rheumatoid arthritis [24], waiting to declare sacroiliitis as a disease manifestation until changes appear on radiographs is a missed opportunity to maximally improve long-term clinical, functional, and radiographic outcomes. Further, radiographs are not without consequences to children and their parents, including anxiety (from extra imaging procedures or false positive results), radiation exposure, and costs. Procedural costs for a single AP pelvic radiograph at our institution is approximately $97.00 for one or two views and $132.00 for dedicated sacroiliac joint views; additional fees are charged for professional interpretation.

Second, imaging studies always need to be clinically correlated, considering both the pretest probability and suspicion for disease. All of the children included in the study had suspected inflammatory sacroiliitis either because of underlying diagnosis or symptoms. The majority of positive MRI studies had normal radiographs but, given clinical suspicion, MRI studies were ordered anyway. At least half of the radiographs considered indicative of sacroiliitis by all raters were accompanied by a normal MRI scan. Further, even when we think the radiograph is truly depicting evidence of joint damage and not a false positive result, the findings do not indicate whether the disease remains active and requires treatment or whether the disease has already burnt out. Therefore, abnormal radiographs are almost always followed by an MRI study. If the pretest suspicion of sacroiliitis is high enough that we are going to order the MRI scan regardless of the radiograph findings, why not save the patient and family from anxiety, radiation exposure, and healthcare dollars and jump straight to the MRI?

## Conclusions

This is a systematic comparison of the utility of radiographs and MRI in children with suspected inflammatory sacroiliitis. Our results demonstrate very limited utility of radiographs for the detection of sacroiliitis. In parts of the world where MRI is readily available, obtaining or requiring radiographs prior to MRI is an antiquated practice.

## Abbreviations

AP: Anterioposterior
ASAS: Assessment of Spondyloarthritis International Society
FABER: Flexion abduction external rotation
IQR: Interquartile range
JSpA: Juvenile spondyloarthritis
MRI: Magnetic resonance imaging
PACS: Picture archiving and communication system
REDCap: Research Electronic Data Capture
SIJ: Sacroiliac joint
SIS: Sacroiliac joint inflammation score
SPARCC: Spondyloarthritis Research Consortium of Canada
SSS: Sacroiliac joint structural score
